# Potentially avoidable hospitalization for asthma in children and adolescents by migrant status: results from the Italian Network for Longitudinal Metropolitan Studies

**DOI:** 10.1186/s12889-020-09930-9

**Published:** 2020-12-04

**Authors:** Laura Cacciani, Cristina Canova, Giulia Barbieri, Teresa Dalla Zuanna, Claudia Marino, Barbara Pacelli, Nicola Caranci, Elena Strippoli, Nicolás Zengarini, Anteo Di Napoli, Nera Agabiti, Marina Davoli

**Affiliations:** 1 Dipartimento di Epidemiologia del Servizio Sanitario Regionale del Lazio, Asl Roma 1, Roma, Italy; 2 Dipartimento di Scienze Cardio-Toraco-Vascolari e Sanità Pubblica, Università di Padova, Padova, Italy; 3 Agenzia Sanitaria e Sociale Regionale, Emilia-Romagna, Bologna, Italy; 4 S.C. a D.U. Servizio Sovrazonale di Epidemiologia - ASL TO3, Torino, Italy; 5 Istituto Nazionale Salute Migrazioni e Povertà – INMP, Roma, Italy

**Keywords:** Immigrants, Children and adolescents, Potentially avoidable hospitalization, Asthma, Healthcare disparities, Longitudinal study, Italy

## Abstract

**Background:**

Global migration toward Europe is increasing. Providing health assistance to migrants is challenging because numerous barriers limit their accessibility to health services. Migrants may be at a greater risk of developing asthma and receiving lower quality healthcare assistance than non-migrants. We aim to investigate whether immigrants as children and adolescents have higher rates of potentially avoidable hospitalization (PAH) for asthma compared to Italians.

**Methods:**

We performed a retrospective longitudinal study using six cohorts of 2–17-year-old residents in North and Central Italy from 01/01/2001 to 31/12/2014 (*N* = 1,256,826). We linked asthma hospital discharges to individuals using anonymized keys. We estimated cohort-specific age and calendar-year-adjusted asthma PAH rate ratios (HRRs) and 95% confidence intervals (95%CIs) among immigrants compared to Italians. We applied a two-stage random effect model to estimate asthma PAH meta-analytic rate ratios (MHRRs). We analyzed data by gender and geographical area of origin countries.

**Results:**

Three thousand three hundred four and 471 discharges for asthma PAH occurred among Italians and immigrants, respectively. Compared to Italians, the asthma PAH cohort-specific rate was higher for immigrant males in Bologna (HRR:2.42; 95%CI:1.53–3.81) and Roma (1.22; 1.02–1.45), and for females in Torino (1.56; 1.10–2.20) and Roma (1.82; 1.50–2.20). Asthma PAH MHRRs were higher only among immigrant females (MHRRs:1.48; 95%CI:1.18–1.87). MHRRs by area of origin were 63 to 113% higher among immigrants, except for Central-Eastern Europeans (0.80; 0.65–0.98).

**Conclusion:**

The asthma PAH meta-analytic rate was higher among female children and adolescent immigrants compared to Italians, with heterogeneity among cohorts showing higher cohort-specific PAH also among males, with some differences by origin country. Access to primary care for children and adolescent immigrants should be improved and immigrants should be considered at risk of severe asthma outcomes and consequently targeted by clinicians.

## Background

International migration is increasing and health assistance to migrants is challenging because of barriers which often limit accessibility to healthcare services, even when health systems include assistance to migrants [1]. In Italy, the number of immigrants in 2018 was 8.5% of the resident population [2]. The main reasons for migration to Italy among non-EU citizens were job opportunities and family reunification (88% in 2001, 74% in 2013, and 64% in 2014, when residence permits for international protection increased in number following the 2011 Arab Spring and the humanitarian emergency of 2014–2015 [3]). Most immigrants came from low-income countries attracted by the high demand for unskilled jobs, especially in the sectors of construction and domestic services [4].

While the prevalence and risk of some non-communicable diseases in migrant groups living in Europe have been estimated, especially for cardio-vascular diseases, diabetes, and cancer, less and fragmentary evidence is available for other non-communicable diseases, such as asthma [5]. The burden of asthma is relevant because it also affects children and adolescents, and the prevalence of the disease is increasing in many high-income countries. It has been estimated that around 358 million individuals in the world were affected by asthma in 2015 [6]. In Italy, the prevalence of lifetime asthma was estimated to be 9.3% in children and 10.3% in adolescents [7]. However, these measures for Italy might be an underestimation, as there is likely to be a pool of asthma patients yet unrecognized by healthcare [8].

The complex relationship between individual and environmental factors on asthma occurrence in children, including maternal exposure to negative factors during pregnancy, was highlighted in several observational studies in Europe [9–11]. Migration likely plays an additional role in the pathogenesis of asthma in children, but the mechanisms are still not clear. Immigrants may be at a higher risk of developing asthma due to the exposure to new pollutants in the host countries, changes of lifestyles, and deprived housing. A study conducted in Italy in 2007 found that the prevalence of symptoms in childhood and adolescent asthma among immigrants was actually lower than among native children when the permanence in the country was less than 5 years; although for longer time periods, the prevalence increased up to the level found among native children, suggesting exposure to environmental risks might be a factor [12].

Asthma is one of the conditions for which hospitalization can be considered avoidable, through timely and effective preventive care and early disease management, referred to as ambulatory care sensitive conditions (ACSCs) [13, 14]. Disease prevention and monitoring of chronic conditions are key points at the primary care level, and potentially avoidable hospitalization (PAH) is frequently used as a quality indicator of primary care delivery [15]. Hospital admissions for patients with chronic disease, such as asthma, are reported within health system performance frameworks of European countries for healthcare quality assessment [16, 17].

To the best of our knowledge, there are no studies on PAH for chronic conditions disparities by migrant status [18] in Italy, except for a recent study focusing on PAH for diabetes mellitus [19]. That study reported that immigrants are at a higher risk of experiencing PAH than Italians, suggesting that they may have less access to and lower quality of primary care.

Given the barriers encountered by immigrants to the provision of healthcare, including primary care [20], our hypothesis is that the diagnosis of asthma and adherence to therapy during childhood and adolescent age may be reduced. This would be a potential cause for immigrants being at higher risk of disease complications, as well as for increased asthma severity compared to Italians; this phenomenon may be mirrored in the excess PAH for asthma among immigrants. We have focused on children and adolescents because in this segment of the population asthma is the most common chronic disease [21]. To verify our hypothesis, we compared PAH for asthma among children and adolescents by migrant status during the period 2001–2014, using population-based cohort data from six cities in North and Central Italy included in the Italian Network of Longitudinal Metropolitan Studies (IN-LiMeS) [22–24]. Our objective is to examine whether immigrant children and adolescents living in Italy have higher rates of PAH for asthma compared to Italians.

## Methods

### Study design and setting

This is a retrospective longitudinal study based on dynamic cohorts of residents in six cities participating in the IN-LiMeS [22–24]: Torino, Venezia, Reggio Emilia, Modena, Bologna, and Roma. Each cohort represents one city. Data from the participating cohorts can be analyzed together to create a pattern of outcomes in relation to sociodemographic characteristics in Italy. For this analysis, participants were recruited and followed-up between 01/01/2001 (21/10/2001 for Torino) and 31/12/2013 (31/12/2014 for Venezia).

### Participants

**Fig. 1:**
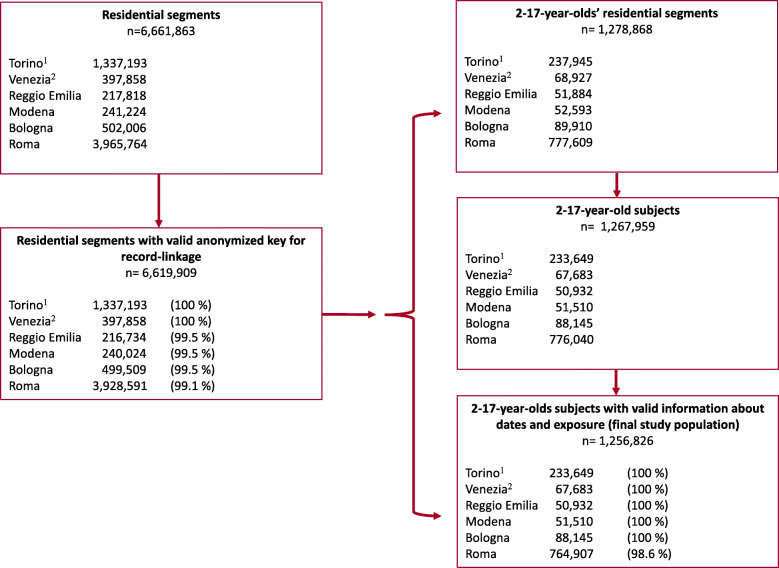
Selection of the study population by cohort. Years 2001–2014. ^1^ Entry in the cohort: 21/10/2001. ^2^ End of follow-up: 31/12/ 2014

The participants in the study were all 2–17-year-old individuals registered as residents in the Population Registries of at least one of the six cities included in the study during the follow-up period (*N* = 1,256,826). The age selection followed the criteria of the Agency for Healthcare Research and Quality (AHRQ), and the case definition for pediatric asthma [25, 26]. Non-resident immigrants were not included, while refugees and asylum seekers were included after having obtained a permit for residence. In 2015, about 86% of immigrants in Italy were registered as resident [27]. To calculate person-time-at-risk, we built datasets containing all time periods of residence for everyone who resided in one of the aforementioned cities for at least 1 day during the study period (residential segments). Net person-years (PYs) at risk were calculated excluding time periods that occurred between emigration and re-immigration in each city. The entry date for the cohort was the registration date in the Population Register from birth or immigration after 01/01/2001 (21/10/2001 for Torino), while the exit date was for the 18th birthday, emigration, death or end of the study (31/12/2013 or 31/12/2014 for Venezia) whichever was the less recent. Record-linkage procedures between demographic and hospital data were performed deterministically using anonymous keys to guarantee the privacy of individuals. Detailed description of the method can be found elsewhere [22–24]. Figure 1 displays the total number of residential segments; the number of segments with valid anonymous keys for record-linkage to hospital data; the number of eligible residential segments and subjects, and the final study population with valid information on entry and exit dates and exposure for each cohort.

### Variables, data sources and measurement

#### Outcome

The outcome variable was the number of all possible PAHs (inpatient hospital discharges) for asthma that occurred during the follow-up time period, within or outside the region where each city is located (the regions are large territorial units, with 4,4 to 5,9 million inhabitants). The hospital discharge records include up to six diagnostic codes, reported according to the International Classification of Diseases, Ninth Revision, Clinical Modification (ICD-9-CM). To define PAH for asthma, we used the AHRQ definition of Pediatric Quality Indicators (PDIs), which focus on preventable hospitalizations among pediatric patients. PDIs are measures of potentially preventable hospitalizations for ACSCs, also called potentially avoidable hospitalizations [18], that is, “inpatient stays for treating ambulatory care sensitive conditions (ACSCs) that evidence suggests may be avoidable, in part, through timely and quality primary and preventive care” [28]. Among the indicators, we chose the indicator number 14 “Asthma Admission Rate”. For the selection of cases, we used the AHRQ Technical Specifications [26]. Thus, we selected cases with the ICD-9-CM code 493 in the principal diagnosis. Exclusions in secondary diagnoses for cystic fibrosis and anomalies of the respiratory system were also applied (ICD-9-CM codes 277.0, 516.6, 747.2, 748.3–9, 750.3, 759.3, 770.7). In addition, we applied the other AHRQ criteria which exclude the following cases: transfer from a hospital (different facility), a Skilled Nursing Facility or Intermediate Care Facility, or from another health care facility; cases of pregnancy, childbirth, and puerperium (Major Diagnostic Criteria 14). The source of data is the Hospital Discharge Record which is collected through the Hospital Information System of the region where each city is located.

#### Exposure and other risk factors

The exposure variable was the migrant status as measured by the citizenship of High Migratory Pressure Countries and related geographical areas of origin (HMPCs, immigrants: Central-Eastern Europe, North Africa, Sub-Saharan Africa, Asia -except for Israel and Japan-, and Central and South America) or High Developed Countries (HDCs: foreign countries except those included in the HMPCs) as contrasted with Italians. We used the birthplace as a proxy of the migrant status for the population of Roma until 2007 because the information on citizenship was not available. We considered age (in years) and calendar year as confounding variables, while gender and cohort as strata variables. The source of data was the Population Register of each city participating in the study, integrated with the dataset of Hospital Discharge Records. We excluded records with missing data on migrant status, age, calendar year, and gender (overall, less than 1.4%).

### Statistical analysis

We focused the statistical analysis only on the comparison between Italians and immigrants coming from HMPCs because the latter represent the majority of vulnerable immigrants in relation to health assistance and because the percentage of immigrants from HDCs was very low (it ranged between 0.13% in Reggio Emilia and 0.90% in Roma). We calculated crude (CHRs) and direct age-standardized hospitalization rates (SHRs) using the 2011 Italian census population. CHRs and SHRs were calculated from the total population for each cohort as well as for the total study period (2001–2014). Then, we estimated age and calendar-year-adjusted hospitalization rate ratios (HRRs), and the related 95% confidence intervals (95%CIs) for immigrants from HMPCs compared to Italians, using the negative binomial regression, which allowed us to model the number of occurrences of an event when it has overdispersion, with PYs as the exposure. We stratified the analysis by gender because males were found to have higher asthma prevalence than females during childhood, but lower prevalence and better outcomes at later ages [29]. In addition, we stratified the analysis by cohort to acknowledge any unmeasured contextual factors of the cohort that could have influenced the associations. To provide a global measure of effect we performed a random effect Individual Participant Data (IPD) meta-analysis [30]. Thus, we estimated meta-analytic hospitalization rate ratios (MHRRs) and related 95%CIs. We performed the Cochran heterogeneity test to assess whether the differences observed in the effect measures of each cohort were due to chance and, to quantify such heterogeneity, we calculated the I^2^ statistic, which describes the percentage of variability across studies [31]. We also estimated MHRRs by geographical area of origin; but in this case we could not stratify by gender because in some combinations of cases by gender and area of origin there were no observations. We considered two-sided *p*-values less than 0.05 as statistically significant. We managed the data using the software SAS Enterprise guide 7.1 and analyzed the data using the Stata software, version 13, commands *nbreg*, and *metan* (with options *randomi*).

### Linkage

The study included person-level-data-linkage across Population Register and Hospital Discharge Records.

The data were linked using anonymized keys and then analyzed in aggregate form. We assessed the linkage quality by calculating the record linkage percentage that was greater than 99.1% (Fig. 1).

## Results

### Participants and descriptive data

Figure 1 displays the selection of the population included in the study. We selected 1,256,826 2–17-year-old subjects during the follow-up time period (18.6% in Torino, 5.4% in Venezia, 4.1% in Reggio Emilia, 4.1% in Modena, 7.0%, Bologna, and 60.9% in Roma) (Table A1, additional material) who contributed with 8,070,415 PYs. The percentage of individuals coming from HMPCs was 13.7%, with percentages ranging between 11.0% in Roma and 22.2% in Reggio Emilia, while those coming from HDCs were only 0.7%. Among the immigrants, the highest percentage of individuals was observed among Central-Eastern Europeans (41.3%) and the lowest among Sub-Saharan Africans (7.3%). Among the participants, there was the same proportion of males and females. The mean age calculated over the whole study period was 9.3 years. The number of subjects enrolled was 608,240 in 2002 and 673,964 in 2013 (2002 and 2013 were the first and last calendar years with complete follow-up for all the cohorts).

### Description of hospital data

During the study period, 3787 hospital discharges for asthma were registered among 2–17-year-old participants, 3304 among Italians (87%) and 471 (12%) among immigrants from HMPCs. Among immigrants, the highest percentage of discharges for asthma was observed for subjects from Asia (38%) and the lowest for those from Sub-Saharan Africa (11%). Males showed higher percentages of hospitalization than females (60% among Italian males and 55% among immigrant males). The mean age at admission was 8.2 years (8.3 among Italians and 5.0 among immigrants). The number of hospitalizations ranged from 426 in 2002 to 182 in 2013, with a clear decrease among Italians and a steady pattern among immigrants.

### Asthma PAH rates

Age-standardized rates (per 1000 PYs) ranged from 0.18 (95% CIs:0.13–0.24) in Modena to 0.54 (0.46–0.61) in Venezia among Italians, and from 0.14 (0.35–0.54) in Modena to 0.68 (0.60–0.75) in Roma among immigrants. Among Italians, gender specific rates were always higher among males, from 0.19 (0.12–0.27) in Modena to 0.63 (0.57–0.70) in Torino, compared to females whose rates were from 0.17 (0.10–0.25) in Modena to 0.48 (0.38–0.59) in Venezia. Among immigrants, rates ranged from 0.05 (0.00–0.14) in Modena to 0.80 (0.46–1.13) in Reggio Emilia among males, and from 0.24 in Modena (0.03–0.45) and Bologna (0.06–0.42) to 0.66 (0.58–0.79) in Roma among females (Table 1).

**Table 1:** Discharges, PYs, crude (CHR) and age-standardized (SHR) asthma PAH rates by citizenship, cohort, and gender

Cohort	Country/area of origin	Males				Females				Total			
		N	PYs	CHRx1000	SHRx1000	N	PYs	CHRx1000	SHRx1000	n	PYs	CHRx1000	SHRx1000
Torino^a^	Italy	357	608,326	0.59	0.63	225	574,698	0.39	0.42	582	1,183,025	0.49	0.53
	HMPCs	47	90,076	0.52	0.48	40	84,951	0.47	0.42	87	175,028	0.50	0.45
Venezia^b^	Italy	115	211,898	0.54	0.59	90	196,875	0.46	0.48	205	408,774	0.50	0.54
	HMPCs	7	25,629	0.27	0.29	10	23,315	0.43	0.43	17	48,944	0.35	0.35
Reggio Emilia	Italy	71	133,779	0.53	0.55	51	127,099	0.40	0.42	122	260,879	0.47	0.49
	HMPCs	22	26,816	0.82	0.80	10	24,695	0.40	0.36	32	51,511	0.62	0.59
Modena	Italy	26	144,965	0.18	0.19	21	136,198	0.15	0.17	47	281,163	0.17	0.18
	HMPCs	1	19,375	0.05	0.05	5	18,202	0.27	0.24	6	37,577	0.16	0.14
Bologna	Italy	91	242,597	0.38	0.40	54	228,716	0.24	0.25	145	471,313	0.31	0.32
	HMPCs	25	29,964	0.83	0.78	7	28,049	0.25	0.24	32	58,013	0.55	0.52
Roma^c^	Italy	1345	2,380,197	0.57	0.61	858	2,246,522	0.38	0.42	2203	4,626,719	0.48	0.52
	HMPCs	155	223,863	0.69	0.68	142	207,134	0.69	0.66	297	430,998	0.69	0.68

*HMPCs* High Migratory Pressure Countries, *PYs* Person-years

^a^Entry in the cohort: 21/10/2001

^b^End of follow-up: 31/12/ 2014

^c^We used the birthplace for individuals residing in Roma until 2007

### Results from the negative binomial regression and meta-analytic results

Compared to Italian counterparts, excess-age and calendar-year-adjusted asthma PAH rates were found only among immigrant males in Bologna (HRR:2.42; 95%CI:1.53–3.81) and Roma (1.22; 1.02, 1.45), and among immigrant females in Torino (1.56; 1.10–2.20) and Roma (1.82;1.50–2.20). Heterogeneity was observed among areas of origin in the HMPCs group, though estimates were imprecise, due to low or null numbers of cases.

**Fig. 2:**
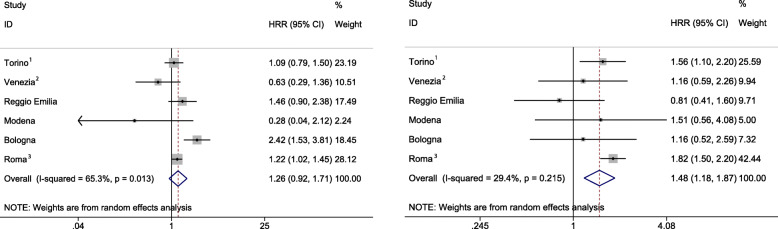
Age and calendar-year-adjusted asthma PAH HRRs (HMPCs vs Italy) by cohort and gender, and MHRRs. ^1^ Entry in the cohort: 21/10/2001. ^2^ End of follow-up: 31/12/ 2014. ^3^ We used the birthplace for individuals residing in Roma until 2007

Meta-analytic results showed a higher asthma PAH rate among immigrants compared to Italians among females only (MHRR:1.48; 95%CI:1.18–1.87). Results among males also showed a higher asthma PAH rate among immigrants compared to Italians (MHRR:1.26; 95% CI:0.96–1.71), though not statistically significant. Also, the statistic I^2^ showed a substantial level of heterogeneity across studies for males (65.3%; *p* = 0.013) (Fig. 2).

### Results by geographical area of origin

Compared to Italians, 63 to 113% meta-analytic excess overall (males and females) PAH was found for all the areas of origin, except for immigrants coming from Central-Eastern Europe (MHRR:0.80; 95% CI:0.65–0.98), with some heterogeneity across cohorts (Table 2).

**Table 2:** Random effect meta-analytic asthma PAH rate ratios (MHRR) adjusted for age and calendar-year (reference: Italy)

Country/area of origin	MHRR	95%CI		I squared	*P*-value Cochran
		Lower limit	Upper limit		
Central-Eastern Europe	0.80	0.65	0.98	0%	0.566
Northern Africa	1.63	1.26	2.10	8%	0.365
Sub-Saharan Africa^a^	2.13	1.48	3.07	29%	0.232
Central-Southern America	1.95	1.52	2.49	0%	0.878
Asia	1.66	1.42	1.94	0%	0.502

^a^The number of cohorts with HRR > 0 included in the meta-analysis was 6 except for Sub-Saharan Africa, which was 5

## Discussion

Using an IPD meta-analytic approach, this multi-cohort longitudinal study showed higher meta-analytic PAH rate for asthma among female children and adolescent immigrants from HMPCs compared to Italians. Cohort specific results also showed some heterogeneity across the cohorts and differences also among male immigrants and Italians. Indeed, we found higher cohort-specific rates for immigrant females in Torino and Roma; and also for immigrant males in Bologna and Roma. The meta-analytic analysis by area of origin showed higher hospitalization for immigrants compared to Italians, except for those coming from Central-Eastern Europe. The excess was marked among Sub-Saharan Africans.

There are no studies on PAH for asthma focusing on the comparison between immigrants and non-immigrants in Italy. Very few such PAH studies are available in other European countries, even though these studies are often based on the concept of ethnicity or minority group [18, 32]. Although the concepts of “migrant group” and “ethnic minority group” differ substantially, they partially overlap in countries where the migration process is relatively recent, and particularly in some European countries [33]. Among the European studies, a retrospective cohort study conducted in Scotland over a 9-year study period found ethnic variations in first asthma hospitalization or death from asthma [34]. The study population included all ages. One explanation of the results suggested by the Authors was the variation in the quality of care provision across different ethnic groups, which may also hold true for our results. Also, a systematic review conducted in the UK, found a disparity between the reduced prevalence of asthma among some ethnic minorities and a higher asthma-related use of health services [35]. The Authors concluded that ethnic minorities probably receive different amounts of primary care compared to white patients.

If we consider evidence from the USA, we find studies documenting racial disparities in hospitalizations for asthma in children and young adults, with black people showing increased hospitalization rates compared with white patients [36–38]. Another study found racial and ethnic differences in asthma prevalence despite access to care, with African American and Hispanic children more likely to have PAH for asthma and asthma-related emergency department visits, and less likely to visit a specialist compared with white children [39]. Various factors have been suggested to explain the differences observed, including the quality of medical care, home environment, and genetic differences. However, these studies conducted in the USA, have a different exposure variable and migration patterns are different compared to Italy, making the comparison to Italy sub-optimal.

### Interpretation and generalizability

As among females, immigrant children and adolescents have higher PAH than Italians, while among males they show higher PAH than Italians in the cohorts of Bologna and Roma. A possible interpretation of these findings is that they might reflect a combination of increasing asthma prevalence after immigration, more severe asthma symptoms, a lower ability to manage asthma symptoms and lower care; in addition to more barriers in accessing primary care and increased utilization of emergency units. Barriers may occur at patient level, provider level and system level [40], and may depend on problems with language (such as difficulties to describe an illness or to understand medical advice), bureaucratic aspects (e.g. lack of proper documentation to access the health service), cultural (e.g. different recognition of symptoms or perception of an illness), or organizational (e.g. difficulties to combine own-work-time with opening-time of health provider) [1]. In addition, immigrants may experience specific barriers depending on the local context where they live, which may differently affect the use of health services. In a systematic review on the development of asthma and allergic diseases in relation to international immigration, which involves moving from low income to Western countries, the Authors found that although the prevalence of asthma among immigrants was lower than among non-immigrants, it increased steadily with length of residence [41]. This may suggest that environmental factors are involved in the development of asthma among the usually healthy immigrants, and that barriers accessing primary care [20] may be related to the observed increased rate of hospitalization. Also, the severity of the disease, which was found to be related to the ethnic background [42], may be involved to explain the higher hospitalization for asthma. In the United States, asthma attacks were found higher among African American children [43], and the risk of ED visits were found higher among non-whites and Hispanics, compared to non-Hispanic whites [44]. Another explanation may be that PAH is the consequence of a low ability to manage asthma among immigrants. Racial and ethnic minorities disparities for pediatric asthma care were observed in the United States as consequences of the interactions between the patient family and the healthcare provider [45].

The higher meta-analytic PAH for asthma among female children and adolescent immigrants compared to non-immigrants, but not males, may be explained by factors such as gender differences of severity and prevalence at different ages [29], or of compliance to treatment and awareness. However, we also found higher meta-analytic PAH among immigrant males compared to non-immigrants, although not statistically significant, as well as higher cohort-specific PAH in some cohorts. Alternative explanations, such as some heterogeneity of the results among the cohorts and the low number of events in some cohorts, can be invoked. Therefore, further analysis is necessary to better examine and give interpretation for the gender-specific patterns observed.

We also found that Sub-Saharan Africans had the highest rate of PAH for asthma. Studies conducted in Italy on different health outcomes, such as mortality [23] or perinatal outcomes [46], showed vulnerability of immigrants from Sub-Saharan Africans. A study conducted in the United States found a consistent association of African ancestry with asthma risk [47]. Our interpretation is that socioeconomic, environmental, and genetic factors may all play a role. On the other hand, we found lower risks for immigrants from Central-Eastern countries which may depend on a higher integration of this population in Italy. Further studies are required to explain the variability observed among immigrant groups.

Finally, the results suggest some differences among the cohorts. We think that the differences may be related to unmeasured individual and contextual factors, such as the severity and prevalence of the disease, the composition of the immigrant population and their health literacy, or the accessibility to healthcare services in the local context. However, caution is needed in the interpretation as they may also be related to the very low number of events in some cohorts.

It is posited that some advantages of the present study, particularly in the creation and use of longitudinal studies, are such that they give value and validation to the Authors’ interpretation. The study encompasses 13 years and provides sufficiently enough cases to measure the associations. In addition, the study is conducted using data from six cohorts of all residents in cities located in the North and Central Italy. This data may mirror the effect of local policies on healthcare assistance to different migratory flows, and consequentially, on the amounts of prevailing PAH for asthma. The use of standardized archives of data and shared procedures for data analysis guarantee the internal validity of the results. The inclusion of cities located in the North and Central Italy, where the number of immigrants is high, support the generalizability of results to other Italian cities and to cities of other European countries with universal health systems and immigration from HMPCs.

### Limitations

A limit of our study is that we could not include relevant risk factors which might confound the associations or act as effect modifiers. For example, the time period of residence may have a bidirectional effect on asthma because on the one hand it may increase health literacy, and thus the ability to access primary care, and on the other hand it may increase the risk of the onset of asthma and episodes as a consequence of exposure to environmental factors [12]. Another relevant factor is the socioeconomic status which is negatively associated to an increased risk of asthma [48]. The socioeconomic status might also be an effect modifier of the association between the migrant status and asthma. Indeed, there is evidence that disadvantaged migrants have better health than advantaged non-migrants, an effect known as the epidemiological paradox [49, 50]. Finally, despite the fact that we expected the prevalence of asthma to be different among the groups compared [12, 51], which may affect the risk of hospitalization, the prevalence estimate was not available and therefore it was not possible to consider its effect on the results.

We must mention two relevant aspects concerning misclassification and its possible related information bias. Firstly, we used the birthplace instead of citizenship as a proxy measure of the migrant status for people residing in Rome before the year 2007, as citizenship at that time was not available. Thus, the offspring of immigrants in Italy would be classified as Italians, even though they had a migrant background. In 2007, 11.3% of those born in Italy had foreign parents [52]. Conversely, very few Italians were born in another country (less than 0.3%) [53]; and while it was difficult to estimate the effect of this potential misclassification, we expect that it would not have changed the direction of the association observed, because the analysis was based on a very large population. It may be useful to consider that in another study, where we calculated cause-specific and overall hospitalization rates using data from the same cohorts, we found the results for Rome to be more similar to those of all the cohorts included, after restricting the analysis to the years 2008–2013, compared to 2001–2013. However, the results were not much different from the results over the whole study period 2001–2013 [24].

Secondly, the migrant status may be misclassified for those individuals who acquired Italian citizenship during the study period. However, the percentage of individuals obtaining Italian citizenship was low due to the legislation in force, the *ius sanguinis*, which requires many years of residence in Italy to be eligible for Italian citizenship. According to the Italian National Institute of Statistics, about 135,814 non-EU immigrants acquired the Italian citizenship in 2017, representing only 3,7% of the non-EU immigrants living in the country [54].

## Conclusion

This is the first study on PAH for a relevant chronic disease such as asthma among children and adolescent immigrants and Italians, based on six cities participating in The Italian Network of Longitudinal Metropolitan Studies representing Central and Northern Italy. The results showed that PAH for asthma was higher among female immigrants from HMPCs compared to the Italian counterparts. Among males, we only observed cohort specific higher PAH for immigrants compared to Italians. We also found differences by geographic area of origin. Since hospitalization for asthma is potentially avoidable, barriers that limit access to and the accessibility of primary care for immigrants should be removed. Also, immigrants should be considered a population at risk of developing severe outcomes due to uncontrolled asthma, and accordingly targeted by clinicians, with specific consideration to their country of origin. The problem of explaining gender specific patterns is a fascinating one, but which the Authors did not have the remit to fully explore. It is something that deserves further study.

## Supplementary Information

**Additional file 1: Table A1.** Study population by citizenship, cohort, and gender.

## Data Availability

The datasets generated and/or analysed during the current study are not publicly available due to stringent legal restrictions regarding privacy policy on personal information in Italy (national legislative decree on privacy policy n. 196/30 June 2003). In addition, due to security aspects, data can be analysed only in a safe place. Researchers may contact the corresponding Author for questions concerning the data, which are available from the Authors upon reasonable request and with permission of the Italian Data Protection Authority.

## Abbreviations

PAH: Potentially avoidable hospitalization
HRRs: Hospitalization rate ratios
95%CIs: 95% confidence intervals
MHRRs: Estimated meta-analytic hospitalization rate ratios
ACSCs: Ambulatory care sensitive conditions
IN-LiMeS: Italian Network of Longitudinal Metropolitan Studies
AHRQ: Healthcare Research and Quality
PYs: Person-years
ICD-9-CM: International Classification of Diseases, Ninth Revision, Clinical Modification
HMPCs: High Migratory Pressure Countries
HDCs: High Developed Countries
CHRs: Crude hospitalization rates
SHRs: Standardized hospitalization rates
IPD: Individual Participant Data
