# Supplementary material for: Potentially avoidable hospitalization for asthma in children and adolescents by migrant status: results from the Italian Network for Longitudinal Metropolitan Studies
Source: BMC Public Health. 2020 Dec 4;20:1858. doi: 10.1186/s12889-020-09930-9 (PMC7716466; doi:10.1186/s12889-020-09930-9)
Supplement: Supplementary file 1 — Additional file 1: Table A1. Study population by citizenship, cohort, and gender. [file 12889_2020_9930_MOESM1_ESM.docx]

Table A1. Study population by citizenship, cohort, and gender.

| Country/area of citizenship |  | Torino^1^ | | | Venezia^2^ | | | Reggio Emilia | | | Modena | | | Bologna | | | Roma^3^ | | |
| --- | --- | --- | --- | --- | --- | --- | --- | --- | --- | --- | --- | --- | --- | --- | --- | --- | --- | --- | --- |
|  |  | Males | Females | Total | Males | Females | Total | Males | Females | Total | Males | Females | Total | Males | Females | Total | Males | Females | Total |
| Italy | n | 98505 | 93040 | 191545 | 29068 | 27212 | 56280 | 20350 | 19222 | 39572 | 21798 | 20393 | 42191 | 37652 | 35512 | 73164 | 327169 | 346205 | 673374 |
|  | col. % | 81.8 | 82.1 | 82.0 | 83.0 | 83.3 | 83.2 | 77.1 | 78.3 | 77.7 | 81.7 | 82.2 | 81.9 | 82.9 | 83.1 | 83.0 | 88.1 | 87.9 | 88.0 |
| HDCs | n | 470 | 451 | 921 | 113 | 106 | 219 | 38 | 31 | 69 | 107 | 110 | 217 | 158 | 181 | 339 | 3466 | 3545 | 7011 |
|  | col. % | 0.4 | 0.4 | 0.4 | 0.3 | 0.3 | 0.3 | 0.1 | 0.1 | 0.1 | 0.4 | 0.4 | 0.4 | 0.3 | 0.4 | 0.4 | 0.9 | 0.9 | 0.9 |
| HMPCs | n | 21406 | 19777 | 41183 | 5848 | 5336 | 11184 | 6002 | 5289 | 11291 | 4789 | 4313 | 9102 | 7617 | 7025 | 14642 | 40526 | 43996 | 84522 |
|  | col. % | 17.8 | 17.5 | 17.6 | 16.7 | 16.3 | 16.5 | 22.7 | 21.6 | 22.2 | 17.9 | 17.4 | 17.7 | 16.8 | 16.4 | 16.6 | 10.9 | 11.2 | 11.0 |
| of which |  |  |  |  |  |  |  |  |  |  |  |  |  |  |  |  |  |  |  |
| *Central-Eastern Europe* | *n* | *10800* | *10111* | *20911* | *2777* | *2583* | *5360* | *1757* | *1586* | *3343* | *1617* | *1497* | *3114* | *2627* | *2484* | *5111* | *15951* | *17178* | *33129* |
|  | *col. %* | *50.5* | *51.1* | *50.8* | *47.5* | *48.4* | *47.9* | *29.3* | *30.0* | *29.6* | *33.8* | *34.7* | *34.2* | *34.5* | *35.4* | *34.9* | *39.4* | *39.0* | *39.2* |
| *Northern Africa* | *n* | *4434* | *3733* | *8167* | *267* | *213* | *480* | *1457* | *1287* | *2744* | *1052* | *853* | *1905* | *900* | *829* | *1729* | *3005* | *3369* | *6374* |
|  | *col. %* | *20.7* | *18.9* | *19.8* | *4.6* | *4.0* | *4.3* | *24.3* | *24.3* | *24.3* | *22.0* | *19.8* | *20.9* | *11.8* | *11.8* | *11.8* | *7.4* | *7.7* | *7.5* |
| *Sub-Saharan Africa* | *n* | *1437* | *1375* | *2812* | *241* | *188* | *429* | *1109* | *947* | *2056* | *1007* | *929* | *1936* | *411* | *398* | *809* | *2172* | *2333* | *4505* |
|  | *col. %* | *6.7* | *7.0* | *6.8* | *4.1* | *3.5* | *3.8* | *18.5* | *17.9* | *18.2* | *21.0* | *21.5* | *21.3* | *5.4* | *5.7* | *5.5* | *5.4* | *5.3* | *5.3* |
| *Central-Southern America* | *n* | *2197* | *2320* | *4517* | *163* | *205* | *368* | *191* | *169* | *360* | *144* | *159* | *303* | *355* | *379* | *734* | *6564* | *6720* | *13284* |
|  | *col. %* | *10.3* | *11.7* | *11.0* | *2.8* | *3.8* | *3.3* | *3.2* | *3.2* | *3.2* | *3.0* | *3.7* | *3.3* | *4.7* | *5.4* | *5.0* | *16.2* | *15.3* | *15.7* |
| *Asia* | *n* | *2521* | *2216* | *4737* | *2395* | *2141* | *4536* | *1488* | *1300* | *2788* | *969* | *875* | *1844* | *3324* | *2935* | *6259* | *12834* | *14396* | *27230* |
|  | *col. %* | *11.8* | *11.2* | *11.5* | *41.0* | *40.1* | *40.6* | *24.8* | *24.6* | *24.7* | *20.2* | *20.3* | *20.3* | *43.6* | *41.8* | *42.7* | *31.7* | *32.7* | *32.2* |
| *Stateless and missing* | *n* | *17* | *22* | *39* | *5* | *6* | *11* | *0* | *0* | *0* | *0* | *0* | *0* | *0* | *0* | *0* | *0* | *0* | *0* |
|  | *col. %* | *0.1* | *0.1* | *0.1* | *0.1* | *0.1* | *0.1* | *0.0* | *0.0* | *0.0* | *0.0* | *0.0* | *0.0* | *0.0* | *0.0* | *0.0* | *0.0* | *0.0* | *0.0* |
| Total |  | 120381 | 113268 | 233649 | 35029 | 32654 | 67683 | 26390 | 24542 | 50932 | 26694 | 24816 | 51510 | 45427 | 42718 | 88145 | 371161 | 393746 | 764907 |

^1^ Entry in the cohort: 21/10/2001.

^2^ End of follow-up: 31/12/ 2014.

^3^ We used the birthplace for individuals residing in Roma until 2007.
